# Thiamine versus placebo in older heart failure patients: study protocol for a randomized controlled crossover feasibility trial (THIAMINE-HF)

**DOI:** 10.1186/s40814-018-0342-0

**Published:** 2018-09-20

**Authors:** Eric Kai Chung Wong, Justin Yusen Lee, Darryl P. Leong, Lawrence Mbuagbaw, Haroon Yousuf, Sabina Keen, Sharon E. Straus, Christopher J. Patterson, Catherine Demers

**Affiliations:** 1 Division of Geriatric Medicine, Faculty of Medicine, University of Toronto, St. Michael’s Hospital, 30 Bond Street, Toronto, Ontario M5B 1W8 Canada; 2 GERAS Centre, Hamilton Health Sciences, McMaster University, 88 Maplewood Ave, Hamilton, Ontario L8M 1W9 Canada; 3 Division of Geriatric Medicine, Department of Medicine, McMaster University, 88 Maplewood Ave, Hamilton, Ontario L8M 1W9 Canada; 4 Division of Cardiology, Department of Medicine, McMaster University, 237 Barton Street E, Hamilton, Ontario L8L 2X2 Canada; 5 Department of Health Research Methods, Evidence, and Impact, McMaster University, 50 Charlton Ave. E, Hamilton, Ontario L8N 4A6 Canada; 6 Division of General Internal Medicine, Department of Medicine, McMaster University, 50 Charlton Ave. E, Hamilton, Ontario L8N 4A6 Canada; 7 Division of Geriatric Medicine, Department of Medicine, McMaster University, 2757 King St. E, Hamilton, Ontario L8G 5E4 Canada; 8 Division of Geriatric Medicine, Faculty of Medicine, University of Toronto, St. Michael’s Hospital, 30 Bond Street, Toronto, Ontario M5B 1W8 Canada

**Keywords:** Heart failure, Thiamine, Placebo, Crossover, Randomized controlled trial, Elderly

## Abstract

**Background:**

Heart failure (HF) is a major cardiovascular disease with increasing prevalence. Thiamine deficiency occurs in 33% of patients with HF. However, the effectiveness of thiamine supplementation in HF is not known.

**Methods:**

In a placebo-controlled randomized two-period crossover feasibility trial, patients age ≥ 60 years with HF and reduced ejection fraction (HFrEF, EF ≤ 45%) will be randomized to thiamine 500 mg oral capsule once daily or placebo for 3 months, then crossed over to the other intervention after a 6-week washout period. The primary outcome is recruitment rate. Secondary outcomes include feasibility and clinical measures. Feasibility outcomes include refusal rate, retention rate, and compliance rate. Secondary clinical outcomes include left ventricular ejection fraction, peak global longitudinal strain measured by echocardiography, N-terminal prohormone of brain natriuretic peptide (NT-proBNP), New York Heart Association (NYHA) functional class, Kansas City Cardiomyopathy Questionnaire (KCCQ) quality of life score, and clinical outcomes (all-cause mortality, HF hospitalizations, and HF emergency room visits).

**Discussion:**

Thiamine is potentially a safe and low-cost treatment for older patients with HFrEF. Results from this study will inform the feasibility of a large clinical trial with clinical endpoints. The findings will be published in a peer review journal and presented at a relevant conference. This study has received full approval from the Hamilton Integrated Research Ethics Board (18-4537) and Health Canada (210603). This trial is funded by the Hamilton Health Sciences New Investigator Grant (15-387) and the McMaster/St. Peter’s Hospital Chair of Aging.

**Trial registration:**

NCT03228030 (ClinicalTrials.gov), registered July 24, 2017.

## Background

Approximately 500,000 Canadians are living with heart failure (HF), with a prevalence of 1–2% [1]. Despite optimal therapy, HF patients experience adverse events, such as hospital admissions and mortality. The 1-year mortality for a hospitalized patient with HF is 33% in Canada [2] and readmission rates are nearly 50% within 1 year of hospitalization, increasing with age [3]. The total health expenditure related to HF is estimated at $3.9 billion per year in Canada [4].

A new direction in HF research emphasizes the role of decreased energy production as a mechanism [5]. Thiamine, also known as vitamin B1, is an essential micronutrient that is required for energy production in the heart [6]. This vitamin is not synthesized in the body, and total body store only amounts to 25–30 mg, which is mostly contained in the skeletal muscles, heart, brain, and kidneys [7]. Thiamine is ingested and absorbed in the jejunum and ileum by active transport. Once absorbed, free thiamine enters the organs, central nervous system, and erythrocytes. Within these tissues and cells, free thiamine is phosphorylated to thiamine pyrophosphate (TPP), which is the biologically active form. Thiamine status is quantified by measuring erythrocyte TPP using high-performance liquid chromatography (HPLC) [8].

Thiamine deficiency is common in patients with HF, with a recent study demonstrating a prevalence of 33% [9]. Thiamine plays an important role in cellular energy production, and its deficiency gives rise to both cardiovascular and neurological consequences. The clinical syndrome of wet beriberi, which is the result of severe thiamine deficiency, resembles HF, with tachycardia, hypotension, and peripheral edema as presenting findings [10]. Heart failure with reduced ejection fraction (HFrEF) is associated with thiamine deficiency in those not taking supplements (49% vs. 24%, *p* = 0.03) [9]. The use of diuretics is also a risk factor for thiamine deficiency, as demonstrated in older studies [11–15]. Age also appears to be an important risk factor, with the prevalence of thiamine deficiency increasing with age [16]. One study of thiamine deficiency in younger patients with HF (*n* = 38; mean age = 47) found only one deficiency case [17].

We propose a randomized placebo-controlled two-period crossover feasibility study of thiamine supplementation in HF. This randomized controlled trial (RCT) is warranted because (i) thiamine deficiency is common in this population and appears related to reduced left ventricular ejection fraction (LVEF); causality is uncertain, so an RCT is necessary to ascertain whether the relationship is causal; and (ii) thiamine supplements have a wide therapeutic window, making this intervention safe and widely applicable. The aims of the present feasibility study are (i) to determine the feasibility of recruitment for an RCT of thiamine supplementation, (ii) to provide data that will permit refinement of sample size estimates for an RCT that is powered for clinical endpoints, and (iii) to optimize trial procedures with a view to conduct a definitive, larger trial.

## Methods

### Design and setting

This is a randomized placebo-controlled two-period crossover feasibility study of thiamine supplementation in HF. The study will include symptomatic, stable older patients with decreased LVEF and recent HF hospitalization in the past 12 months. The chosen population includes patients who are likely to have thiamine deficiency (older HF patients with decreased LVEF) and likely to respond to therapy. Including patients with a recent hospitalization will increase the chances of clinical events, such as recurrent hospitalization or death [18, 19]. A two-period crossover design reduces variance between the treatment and control arms. We will test the feasibility of recruitment at St. Joseph’s Hospital (SJH), and two sites of the Hamilton Health Sciences Corporation (Juravinski Hospital and Hamilton General Hospital) in Ontario, Canada. Eligible patients will be identified on inpatient and outpatient medical units at these sites. Subsequent visits will occur at the Hamilton General Hospital.

### Inclusion criteria

1. Age ≥ 60 years.
2. New York Heart Association (NYHA) class II–IV symptoms.
3. Recent HF-related admission in the past 12 months (defined as HF being reason for admission or HF being a complication during a hospital admission).
4. LVEF ≤ 45% on 2D/3D echocardiography or radionuclide angiography (RNA) in the past 12 months (on optimal therapy).
5. Medically optimized prior to enrolment with angiotensin-converting enzyme inhibitor or angiotensin receptor blocker (± neprilysin inhibitor), β-blocker, and/or aldosterone antagonist at target or maximally tolerated doses.
6. Stable on medications without hospitalization in the past month.

### Exclusion criteria

1. Taking > 2.5 mg/day of thiamine supplement.
2. Unable to swallow study medication.
3. Clinical indication for thiamine supplementation including symptomatic thiamine deficiency (Wernicke’s encephalopathy, severe malnutrition, refeeding syndrome) and heavy alcohol use defined as > 15 standard drinks per week in men and > 10 standard drinks per week in women [20].
4. End-stage renal disease on dialysis.
5. Severe mitral valve disease because this impacts the accuracy of speckle tracking analysis on echocardiography [21].
6. Non-English speaking (unable to complete questionnaires).
7. Unable to provide written consent.
8. Cognitive impairment without a caregiver administering medications.
9. Expected survival < 1 year due to non-cardiac disease.
10. Expected heart transplantation in < 6 months (± left ventricular assistive device).
11. Allergies to the ingredients of the study medication or placebo.

### Screening

Eligible patients will be screened by a research nurse in the Heart Function Clinic at Hamilton General Hospital and by physician investigators on medical inpatient and outpatient units at St. Joseph’s Healthcare Hamilton and sites of Hamilton Health Sciences. Informed consent will be obtained. Older patients with HF often have concomitant cognitive impairment [22]. If a diagnosis of cognitive impairment or dementia is documented or known by the patient/informal caregiver (spouse, family member, or friend involved in patient’s health care), then a caregiver must be available to provide medications daily. If there is any doubt about a participant’s ability to adhere to study medications or protocols, the study nurse will inform the supervising principal investigator, who will make an assessment. Cognitive impairment on its own is not an exclusion criteria as long as there is a reliable way of administering medications (e.g., supervision). However, all participants, even those with cognitive impairment, will be required to provide written informed consent independent of the informal caregiver. If the participant, due to cognitive impairment, cannot comprehend sufficiently to provide informed consent, he or she will be excluded from participation.

### Randomization, enrolment, and crossover arrangement

Identical bottles containing either placebo or thiamine will be labeled by the hospital pharmacy using a computer-generated randomization list (allocation concealment). Only the pharmacy and our statistician (LM) have access to the randomization list. All participants, investigators, and the research nurse will be blinded to group assignments.

Consented patients will be randomized to thiamine or placebo, taken orally once daily for 90 days during the first period. The study medication is *thiamine mononitrate*. Capsules in 500 mg dose are produced by the Accurex Health Care Manufacturing Inc. along with identical placebo capsules containing inert filler. Participants will be instructed not to take the following foods or health products within 2 h of taking the study medication: areca (betel), horsetail (equisetum), raw freshwater fish, and raw shellfish. These foods interfere with thiamine absorption.

Patients will be sequentially assigned to the two groups in a typical AB/BA crossover design [23]. The first group (AB) will be given thiamine (treatment A) for 90 days, followed by a 6-week washout period during which no supplements are given. The patients will then be given placebo (treatment B) for 90 days. The second group (BA) will start with placebo, followed by thiamine. Compliance will be tracked by pill counts and thiamine blood test at each clinic visit (0, 3, 4.5, and 7.5 months). Patients may continue with concomitant medical care.

### Washout period

The rationale for a 6-week washout period is based on previous studies; the half-life of thiamine is 9–18 days based on a physiology study in young healthy men with radiotagged thiamine [24]. Assuming 4–5 half-lives to reach steady-state, drug washout should be completed by 6 weeks. Studies in the 1940s of young women showed that a thiamine-deficient diet caused symptoms of deficiency in about 2–3 weeks [25, 26]. Our study will not restrict thiamine intake through regular diet, so there is no concern of overt symptomatic thiamine deficiency. In HF, there has been one crossover study with thiamine, and a 6-week washout period was used [27].

### Outcomes

As this is a feasibility study, the primary outcome is recruitment rate with a target to recruit 24 subjects in 11 months for study completion in 18 months. The secondary outcomes will include the following feasibility and clinical outcomes. Feasibility outcomes include refusal rate (number of eligible patients who refuse to participate), retention rate (number of enrolled patients completing the study), and compliance rate (measured by proportion of participants taking at least 80% of their study medications).

Secondary clinical outcomes include LVEF and peak global longitudinal strain (GLS, %), NT-proBNP, NYHA functional class, quality of life score, and clinical events, which will all be measured at 0, 3, 4.5, and 7.5 months. GLS and LVEF will be measured on 2D/3D transthoracic echocardiography. The change in GLS and LVEF with supplementation will be used to detect improvement in cardiac function. Change in NT-proBNP reflects HF severity and prognosis [28]. The change in NYHA functional class will be used to track changes in HF symptoms. For quality of life changes, we selected the Kansas City Cardiomyopathy Questionnaire (KCCQ), which is a self-administered disease-specific questionnaire [29]. It has been shown to be responsive to change in large HF trials [30]. For clinical events, all-cause mortality, HF hospitalizations (hospital stay of ≥ 24 h with confirmed diagnosis of HF using the Boston HF criteria), and HF emergency room (ER) visits (hospital stay < 24 h) will be recorded.

### Data collection

All outcome data will be recorded on paper case report forms (CRF) and stored online using REDCap electronic data capture tools hosted at Hamilton Health Sciences [31]. Raw data from 2D/3D echocardiogram machines will be recorded directly into the CRF and secondary echocardiogram variables will be calculated from raw data. The NYHA functional class will be determined clinically by an investigator at each visit using the criteria published by the American Heart Association in 1994 [32]. The KCCQ is a self-administered disease-specific questionnaire [29]. Erythrocyte TPP level will be measured by a local hospital laboratory. NT-proBNP will be measured on a point-of-care device by a technician at the Hamilton Health Sciences laboratory.

### Participant timeline

**Fig. 1:**
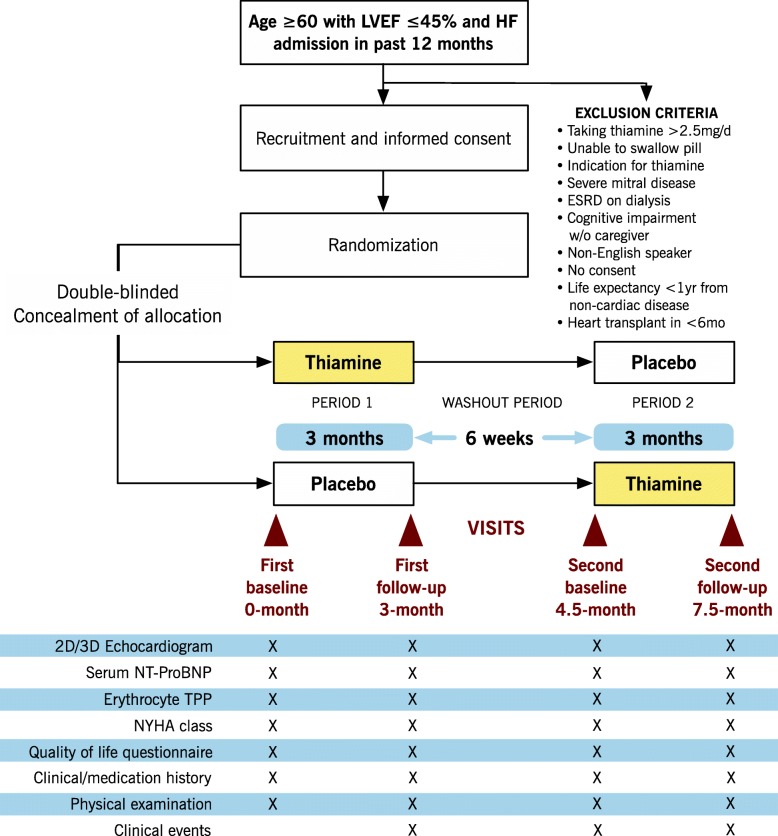
Study flow and assessment intervals

After randomization, patients will attend four study visits: at 0, 3, 4.5, and 7.5 months (Fig. 1). Patients will undergo the following tests at the first visit: (i) 2D/3D transthoracic echocardiogram (determine LVEF and GLS) and (ii) blood sampling for baseline NT-proBNP and erythrocyte TPP (approximately 20 ml of blood). Patients will be instructed not to take any biotin-containing supplements on the day of bloodwork as these can interfere with NT-proBNP assay [33]. Patients will also be asked to complete the KCCQ, followed by an interview with the research nurse for (i) NYHA symptoms, (ii) clinical and medication history, and (iii) physical examination and vital signs. Thereafter, patients will be given a 3-month supply of the study drug to take once a day. Patients will return to the clinic for reassessment and repeat measurements of the above parameters at 3 months. Following a 6-week washout period, patients will attend the 4.5-month visit and the same parameters are measured. The patients will take the alternate drug assignment for another 3 months, followed by a final visit at 7.5 months to measure the parameters. Deaths, hospitalizations, and ER visits will be recorded at each follow-up. The study nurse will look at admission records from local hospitals, including St. Joseph’s Healthcare Hamilton and Hamilton Health Sciences, and ask the patient or caregiver for admissions outside Hamilton. Deaths will be determined from local hospital records or by calling the patient’s emergency contact. If a patient does not attend follow-up visit, contact will be made via phone or caregiver.

### Sample size and recruitment

Using a 95% confidence interval and assuming that 40% of eligible participants will consent to participate in the study, we will need to approach 78 participants in order to recruit 24 (12 per arm). This recruitment rate is based on previous experience with other clinical trials conducted at our site with a similar population. Twelve participants per arm are sufficiently large to inform the feasibility outcomes of the trial and to provide sufficient precision around effect estimates and their variances [34, 35]. Our feasibility trial will be used to inform the following: (i) sample size and power calculations, (ii) expected event rate, (iii) flow of participants, and (iv) dropout rates. This study aims to inform a definitive RCT with clinical endpoint of composite of all-cause mortality and HF hospitalizations.

### Statistical analysis

The process of patient selection and flow throughout the study will be summarized using a flow-diagram. Patient demographics and baseline clinical outcome variables will be summarized using descriptive measures. Data will be analyzed based on the intention-to-treat principle, i.e., data for each participant will be analyzed according to allocation irrespective of whether they complied during the treatment period. Missing data will be replaced using multiple imputation techniques [31]. A secondary per-protocol sensitivity analysis will be conducted for comparison. Although our washout period should be sufficient, we will do a pre-test analysis to check for carry-over effects between the groups. The primary feasibility outcome is reached when 24 patients are recruited within 11 months. Other feasibility thresholds are listed in Table 1. Continuous secondary outcome measures will be tested using analysis of covariance (ANCOVA), with outcome measures adjusted for baseline measures and allocation. McNemar’s test will be used for categorical outcomes. The KCCQ will be analyzed descriptively. All tests will be two-sided, meaning that an effect in either direction will be interpreted. The level of significance is set at *α* = 0.05. Baseline characteristics and results will be reported as means (standard deviation), medians (interquartile range), counts (percentages), 95% confidence intervals, and levels of statistical significance where appropriate. Data will be analyzed using SAS 9.4 (SAS Institute, Cary, NC) and reported according to the CONSORT statement [36].

**Table 1:** Outcome measures

Outcome measure	Scale	Type	Measure	Method of analysis
Feasibility outcomes				
Enrollment rate per year	Ratio	Continuous	Number of participants enrolled per year	Feasibility threshold: 24 patients per year
Refusal rate	Ratio	Continuous	Number of eligible patients refusing to participate	Feasibility threshold: < 40%
Retention rate	Ratio	Continuous	Number of participants completing study	Feasibility threshold: > 80%
Compliance rate	Ratio	Continuous	Number of participants taking study drug appropriately (≥ 80% study drug)	Feasibility threshold: > 90%
Clinical outcomes				
Imaging changes	Ratio	Continuous	Change in global longitudinal strain on speckle tracking echocardiography and ejection fraction	ANCOVA
Biomarker changes	Ratio	Continuous	Change in NT-proBNP	ANCOVA
Functional changes	Ratio	Categorical	Changes in NYHA class	McNemar’s test
Quality of life changes	Ratio	Continuous	Changes on the Kansas City Cardiomyopathy Questionnaire	Descriptive
All-cause mortality	Ratio	Categorical	Difference in mortality between groups	McNemar’s test
Heart failure hospitalizations	Ratio	Categorical	Proportion of hospitalizations due to heart failure	McNemar’s test
Heart failure-related emergency room visits	Ratio	Categorical	Proportion of ER visits due to heart failure	McNemar’s test

## Discussion

With an aging population, the prevalence of HF is expected to rise with a proportional increase in health care costs. Thiamine supplementation is a potentially low-cost and effective intervention to improve cardiac function, particularly in older HF patients with low LVEF.

High-quality RCTs of thiamine supplementation are limited. There are only two published placebo-controlled RCTs in patients with stable HF, both of which were small studies. Shimon et al. [37] randomized 30 HF patients with LVEF ≤ 45% and taking at least 80 mg/day of furosemide to 7 days of IV thiamine vs. placebo, which resulted in a non-statistically significant improvement in LVEF. The use of intravenous thiamine makes the study not easily applicable because this requires either hospitalization or outpatient parenteral therapy, which is not practical.

Schoenenberger et al. [27] completed a two-period randomized crossover study of nine patients with stable HF and LVEF < 40% who were on diuretics to compare oral thiamine 300 mg once daily with placebo for 28 days. There was a statistically (but not clinically) significant improvement in LVEF after 28 days of thiamine therapy (LVEF 32.8% vs. 28.8% placebo, *p* = 0.024). Functional outcomes, including a 6-min walk test and dyspnea score, were unchanged. Limitations of this study include (i) young age of patients (mean age = 56.7 ± 9.2 years), (ii) short treatment duration, and (iii) not all patients were on optimized guideline-driven HF medications. A meta-analysis attempted to pool the results of these two studies and found a significant improvement in LVEF of 3.28% (95% CI 0.64–5.93) for a total of 34 patients [38]. This meta-analysis is limited by the quality of the original studies and the small sample size.

This trial builds on prior knowledge by informing the feasibility of conducting a large RCT with clinical endpoints, as well as exploring changes in cardiac function. Our study is unique from existing trials because (i) it is designed to inform an RCT powered for clinically important endpoints, (ii) it specifically includes patients likely to reach those endpoints, (iii) it uses an extended treatment period, and (iv) it uses sensitive measures of HF that have prognostic significance. Given the low cost of thiamine, high prevalence of HF in our population and possible benefit, this study will guide better HF management with the potential to improve clinical outcomes.

### Trial status

Enrolment begins May 2018.

## Abbreviations

CRF: Case report form
GLS: Peak global longitudinal strain
HF: Heart failure
LVEF: Left ventricular ejection fraction
NYHA: New York Heart Association
RCT: Randomized controlled trial
TPP: Thiamine pyrophosphate
